# Palliative Care Boot Camp Offers Skill Building for Emergency Medicine Residents

**DOI:** 10.5811/westjem.18381

**Published:** 2024-09-06

**Authors:** Julie Cooper, Jenna Fredette

**Affiliations:** * University of Pennsylvania, Department of Emergency Medicine, Philadelphia, Pennsylvania; † ChristianaCare, Department of Emergency Medicine, Newark, Delaware

## BACKGROUND

Emergency medicine (EM) residents routinely care for critically ill patients in both the emergency department (ED) and intensive care units.^1^ Proficiency in primary palliative care skills is essential for all emergency clinicians.^2^ ^,^ ^3^ However, a significant number of residents lack exposure to formal education and training in palliative care.^4^ ^,^ ^5^ Moreover, education and training in palliative care encompasses several Accreditation Council for Graduate Medical Education (ACGME) competencies including system navigation for patient-centered care, understanding the physician’s role in the healthcare system, patient- and family-centered communication, and interprofessional and team communication.^6^

Current curricula addressing primary palliative care skills in EM are notably limited.^7^ ^–^ ^12^ Historically, our residency experienced inconsistencies in the teaching of primary palliative care skills. They were sporadically covered during regular conferences or left to develop organically over time. Furthermore, postgraduate year-2 (PGY-2) residents, who primarily manage seriously ill patients, found themselves engaging in challenging serious-illness conversations with patients and families with little to no training. Recognizing the imperative for more comprehensive education, we introduced a four-week, intensive primary palliative care curriculum specifically tailored for EM PGY-2 residents that was entitled “Palliative Care Bootcamp.”

## OBJECTIVES

The overall objective of the bootcamp was to introduce and strengthen primary palliative care skills among PGY-2 residents at an independent academic medical center. At the end of the curriculum, residents would be able to 1) define the scope of hospice and palliative medicine; 2) understand what primary palliative care skills are for non-specialty trained physicians; 3) recognize ED patients with palliative care needs; 4) implement a hospice evaluation; 5) understand how interdisciplinary teams are involved in the care of seriously ill patients; and 6) build communication skills for discussing goals of care (GOC).

## CURRICULAR DESIGN

The curriculum and assessment were exempt from the institutional review board. Using Kern’s six-step approach to curriculum development, we created an introductory primary palliative care curriculum. An EM faculty member with an interest in palliative care and residency leadership collaborated to develop the curriculum. The residency program endorsed the curriculum as it aligned with a curriculum redesign to include more PGY-specific education.

The curriculum was initially developed in 2017. The interdisciplinary palliative care team at the study institution served as content experts. The team performed a broad review of the residency curriculum and prioritized high-yield topics tailored to the local context. Sessions were scheduled during weekly conference and spanned four consecutive weeks. This schedule allowed for an intensive experience and allowed for rapid skill development. The curriculum is strategically delivered early in the PGY-2 year to leverage residents’ existing experience in caring for seriously ill patients and facilitate meaningful reflection and inquiry.

The curriculum is structured in two phases (Table 1). The first phase spans three weeks and consists of three two-hour sessions. These sessions are dedicated to primary palliative care fundamentals such as an introduction to palliative care, prognosis and trajectory, and non-pain symptom management. Session facilitators included the EM faculty content expert as well as members of the institutional palliative care team, the director of chaplaincy who specialized in family support, the director of palliative care, and the palliative care fellow. Each session encompassed a didactic segment, interactive case-based discussions using scenarios prepared by facilitators or contributed by residents, and opportunities for resident questions.

**Table 1.** The breakdown of palliative care bootcamp sessions by hour detailing the topic, learning objectives, mapping to ACGME^*^ competencies, and the format of the session.

Hour	Topic	Objectives	ACGME competencies	Format/facilitator
1	Intro to primary palliative care in emergency medicine	Define primary palliative care and identify common ED presentations of patients with unmet palliative care needs. Define advance care planning, goals of care, code status and treatment limitations and describe how these are codified in legal and medical documents Interpret a POLST (Physician Orders for Life Sustaining Treatment) form and describe its use in acute care settings	*System navigation for patient centered care* *Physician role in healthcare systems*	Lecture – EM faculty content expert
2	Prognosis and trajectory	Describe four common trajectories of life-limiting illness Define prognosis and describe 3 strategies to assess prognosis in ED patients with serious illness	*Diagnosis,* *treatment, and clinical reasoning*	Case-based learning – EM content expert
3	Chaplain chat	Describe the role of the chaplain in the interdisciplinary care of seriously ill patients in the ED	*System navigation for patient-centered care* *Interprofessional and team communication*	Case-based learning -- chaplain
4	Non-pain symptom management	Choose appropriate first- and second-line treatment for seriously ill patients experiencing nausea and vomiting in the ED Choose appropriate first- and second-line treatment for seriously ill patients experiencing dyspnea in the ED Choose appropriate first- and second-line treatment for seriously ill patients experiencing constipation in the ED	*Pharmacotherapy* *Diagnosis, treatment, and clinical reasoning*	Case-based learning – hospital palliative care specialist
5	Ask a consultant	Describe the role of the HPM clinician in the care of seriously ill patients in the hospital Understand the role of HPM consultation in the emergency department	*Interprofessional and team communication*	Case-based learning -- hospital palliative care specialist
6	Intro to hospice	Describe the scope of hospice services and the settings where it can take place Identify patients who may qualify for hospice and how to get them evaluated Provide goal concordant care to patients enrolled in hospice who present to the ED	*System navigation for patient-centered care* *Physician role in healthcare systems*	Lecture – community hospice medical director
7–10	Serious illness communication workshop (VitalTalk)	Practice skills associated with goals of care conversations with a simulated patient.	*Patient- and family- centered communication*	Simulation and standardized patient skills-based practice –EM content expert

* *ACGME*, Accreditation Council for Graduate Medical Education; *ED*, emergency department; *EM*, emergency medicine; *HPM*, hospice and palliative medicine.

In the final week, residents engaged in a four-hour session in the simulation center. This session was led by the EM content expert who is a trained facilitator with Vital Talk, a national non-profit that promotes evidence-based education in serious-illness communication.^13^ This session involves using a standardized patient. Residents are assigned to a small group and they role-play delivering serious news with EM-based scenarios. This session builds skills around delivering serious news.

The curriculum underwent iterative adjustments informed by informal feedback from both facilitators and residents. Modifications were made based on facilitator availability and interest, resulting in the inclusion or modification of topics, while certain subjects, such as opioid pain management, were removed due to redundancy in other educational settings.

## SURVEY DEVELOPMENT

Before implementing the curriculum, we created a brief, pre-bootcamp survey to assess residents’ prior exposure and familiarity with palliative care. Subsequently, two post-surveys were used to gauge residents’ perceptions regarding the achievement of session-specific goals. We developed the first survey to evaluate the first three weeks of the bootcamp. The initial development collected all potential survey items that were refined through expert consultation. The survey used a five-point Likert scale ranging from 1 (strongly disagree) to 5 (strongly agree). The survey items had been pilot tested and refined in preceding years to ensure question clarity (Appendix 1).

A second survey, which was used for the simulation-based session, prompted residents to rate their self-assessed confidence surrounding the specific skills on conducting GOC conversations covered in the session (Appendix 2). The survey uses a five-point Likert scale ranging from 1 (not very confident) to 5 (very confident).

## IMPACT/EFFECTIVENESS

The curriculum evaluation took place during the 2022 bootcamp. Each session had an average of 8–10 PGY-2 residents, of a total 17 potential participants. Attendance varied from week to week due to excused absences. Participation in both pre- and post-surveys was voluntary. Of the eligible residents, nine (52%) completed the pre-survey, revealing that all but one resident had prior exposure to a palliative care rotation during medical school, and 7 of 9 respondents (77%) reported previous communication skills training during their PGY-1 year.

Post-intervention surveys were collected after each session, with completion rates ranging from 25% (2/8 participants) to 70% (7/10 participants) per session. Notably, all respondents indicated agreement or strong agreement with the achievement of each session’s objectives. For the simulation-based communication session, 88% (8/9) reported increased confidence overall, 88% (8/9) of residents reported increased confidence in responding to strong emotions, and 100% (9/9) reported enhanced confidence in eliciting patient goals and values.

## TIPS FOR SUCCESS/CHALLENGES/LESSONS LEARNED

Several key themes emerged regarding the implementation of a bootcamp curriculum in primary palliative care for EM residents. One notable advantage of this curriculum is its longitudinal format, spanning four consecutive weeks with short intervals between sessions. This structure affords residents the opportunity to practice newly acquired skills while actively working in clinical settings, fostering continuous reflection and refinement of their abilities. Additionally, the curriculum is adaptable and enables its implementation in programs lacking EM palliative care-trained faculty. Programs can use local resources such as institutional palliative specialists, interdisciplinary palliative teams, or several publicly available online resources.^9^ ^,^ ^10^ ^,^ ^14^

However, despite its strengths, our curriculum faces several challenges. Notably, residents unable to attend sessions risk missing valuable educational opportunities, as the curriculum is not repeated during the academic year. Moreover, limited opportunities for ongoing skill acquisition and feedback outside scheduled sessions may hinder residents’ ability to fully integrate palliative care principles into their practice. Furthermore, individual programs may be unwilling to invest 10 hours of curriculum to this specific topic and skillset. Lastly, while there was no cost for the simulation time and standardized patients at the study institution, there may be cost associated with this in other programs and this must be considered.

Furthermore, while participants expressed satisfaction with the curriculum, the outcomes data lack the rigor necessary to definitively establish its success. The impact of this curriculum on long-term knowledge or clinical behavior within the ED remains uncertain. It will be important to conduct more formal assessments of the curriculum objectives and to evaluate its application in the clinical setting.

## CONCLUSION

As the role of primary palliative care in emergency medicine continues to evolve, there is a growing need to integrate these essential skills and concepts into all EM residencies. The bootcamp format has proven to be a valuable educational tool in our program, and its effectiveness warrants further exploration and dissemination within the broader EM community.

## Supplementary Information





