# Supplementary material for: Palliative Care Boot Camp Offers Skill Building for Emergency Medicine Residents
Source: West J Emerg Med. 2024 Sep 6;25(6):913–6. doi: 10.5811/westjem.18381 (PMC11610741; doi:10.5811/westjem.18381)
Supplement: Supplementary file 1 [file wjem-25-913-s001.docx]

Appendix 1

Pre-Course Survey Questions (Non-Simulation)

1. Which of the following have you taken as a medical student or resident?

- Palliative Care clinical rotation
- Hospice clinical rotation
- Communication skills course (VitalTalk, SPIKES, death notification, goals of care conversations)
- Other (free text)

1. What is one palliative care skill or topic you would like to cover this month? (free text)

Post-Course Survey Questions

Session 1 Survey

(Likert Scale 1=strongly disagree, 5=strongly agree)

1. After this session, I can better identify ED patients with unmet palliative care needs.
2. After this session, I can better interpret a DMOST form and convert it into ED/hospital orders (DNR, treatment limitations, etc.)
3. After this session, I can better identify the appropriate surrogate decision maker for a patient who lacks capacity.
4. I found the “Palliative Care 101” session to be valuable to my training.
5. I found the “Prognosis and Trajectory” session to be valuable to my training.
6. “Any feedback from today’s session is welcome here.” (free text)

Session 2 Survey

(Likert Scale 1=strongly disagree, 5=strongly agree)

1. After this session, I can better identify the role of the chaplain on a multidisciplinary care team for seriously ill patients.
2. After this session, I can identify first and second line treatments for seriously ill patients experiencing nausea and vomiting in the emergency department.
3. After this session, I can identify first and second line treatments for seriously ill patients experiencing constipation in the emergency department.
4. After this session, I can identify both pharmacologic and non-pharmacologic treatments for seriously ill patients experiencing dyspnea in the emergency department.
5. I found the Chaplain Chat valuable to my training.
6. I found the non-pain symptom management session valuable to my training.
7. “Any feedback from today’s session is welcome here.” (free text)

Session 3 Survey

(Likert Scale 1=strongly disagree, 5=strongly agree)

1. After this session, I can better identify the scope of specialized palliative care services.
2. After this session, I can better identify patients who may benefit from referral to a palliative care specialist.
3. After this session, I can better identify ED patients who may benefit from hospice and how to refer them.
4. After this session, I am more confident partnering with a hospice provider on the care of their patients who are in the ED.
5. I found the Palliative Care Consultant Chat valuable to my training.
6. I found the Hospice 101 session valuable to my training.
7. “Any feedback from today’s session is welcome here.” (free text)

VitalTalk Session Survey

Please rate your self-assessed confidence in the following skills BEFORE today’s session.

Please rate your self-assessed confidence in the following skills AFTER today’s session.

(5 point Likert scale, 1= not at all confident, 5=very confident)

1. Disclosing serious news to a patient or surrogate.
2. Responding to strong emotions that may be elicited during a goals of care conversation.
3. Eliciting a patient’s goals and values as part of a goals of care conversation.
4. Eliciting a patient’s goals and values prior to proposing a specific treatment or limitation.
5. Proposing a medical plan in line with a patient’s stated goals and values

Regarding the timing of this course in your residency training, do you think it was….

1. Much too early
2. A little bit early
3. Right on time
4. A little bit late
5. Much too late
