# Supplementary material for: Palliative Care Boot Camp Offers Skill Building for Emergency Medicine Residents
Source: West J Emerg Med. 2024 Sep 6;25(6):913–6. doi: 10.5811/westjem.18381 (PMC11610741; doi:10.5811/westjem.18381)
Supplement: Supplementary file 2 [file wjem-25-913-s002.docx]

Appendix 2

Simulation Standardized Patient Session Pre-Session Survey

1. Please rate your self-assessed confidence in the following skills BEFORE today’s session.
   1. 5-point Likert scale, 1= not at all confident, 5=very confident
   2. Disclosing serious news to a patient or surrogate.
   3. Responding to strong emotions that may be elicited during a goals of care conversation.
   4. Eliciting a patient’s goals and values as part of a goals of care conversation.
   5. Eliciting a patient’s goals and values prior to proposing a specific treatment or limitation.
   6. Proposing a medical plan in line with a patient’s stated goals and values

Simulation Standardized Patient Session Post-Session Survey

1. Please rate your self-assessed confidence in the following skills AFTER today’s session.
   1. 5-point Likert scale, 1= not at all confident, 5=very confident
   2. Disclosing serious news to a patient or surrogate.
2. Responding to strong emotions that may be elicited during a goals of care conversation.
3. Eliciting a patient’s goals and values as part of a goals of care conversation.
4. Eliciting a patient’s goals and values prior to proposing a specific treatment or limitation.
5. Proposing a medical plan in line with a patient’s stated goals and values
6. Regarding the timing of this course in your residency training, do you think it was
7. Much too early
8. A little bit early
9. Right on time
10. A little bit late
11. Much too late
